# Silver Nanoparticles Induce Mitochondrial Protein Oxidation in Lung Cells Impacting Cell Cycle and Proliferation

**DOI:** 10.3390/antiox8110552

**Published:** 2019-11-14

**Authors:** Reetta J. Holmila, Stephen A. Vance, Stephen Bruce King, Allen W. Tsang, Ravi Singh, Cristina M. Furdui

**Affiliations:** 1 Department of Internal Medicine, Section on Molecular Medicine, Wake Forest University Health Sciences, Winston-Salem, NC 27157, USA; rholmila@wakehealth.edu (R.J.H.); atsang@wakehealth.edu (A.W.T.); 2 Department of Chemistry, Wake Forest University, Winston-Salem, NC 27109, USA; vancesa@wfu.edu (S.A.V.); kingsb@wfu.edu (S.B.K.); 3 Department of Cancer Biology, Wake Forest University Health Sciences, Winston-Salem, NC 27157, USA; rasingh@wakehealth.edu

**Keywords:** silver nanoparticles, protein oxidation, mitochondria, cell cycle

## Abstract

Silver nanoparticles (AgNPs) are widely used nanomaterials in both commercial and clinical biomedical applications, due to their antibacterial properties. AgNPs are also being explored for the treatment of cancer in particular in combination with ionizing radiation. In this work, we studied the effects of AgNPs and ionizing radiation on mitochondrial redox state and function in a panel of lung cell lines (A549, BEAS-2B, Calu-1 and NCI-H358). The exposure to AgNPs caused cell cycle arrest and decreased cell proliferation in A549, BEAS-2B and Calu-1, but not in NCI-H358. The mitochondrial reactive oxygen species (ROS) and protein oxidation increased in a time- and dose-dependent manner in the more sensitive cell lines with the AgNP exposure, but not in NCI-H358. While ionizing radiation also induced changes in the mitochondrial redox profiles, in general, these were not synergistic with the effects of AgNPs with the exception of NCI-H358 and only at a higher dose of radiation.

## 1. Introduction

Engineered nanoparticles, defined as materials having at least one dimension less than 100 nm in size, made of materials, including metals (silver, gold, iron, and others), are increasingly used for industrial application and as components of consumer products [1,2]. Because of their small size and the resulting changes in physical and chemical properties, nanoparticles can potentially be used to treat diseases, including cancer at the subcellular level [3]. However, they may cause adverse health effects that are distinct from the known toxicities of bulk materials [1]. As the use of nanoparticles expands, human exposure in occupational settings, after consumer use, and in a medical application will also increase [2,4]. 

Among metallic nanoparticles, silver nanoparticles (AgNPs) are the most widely used for consumer, industrial, and biomedical applications because of their unique physical, chemical, optical, catalytic, antibacterial properties [4,5,6]. They are manufactured in a wide variety of sizes and forms, including spheres, rods, cubes, wires and triangles [6]. Due to the antimicrobial activity, AgNPs are used in several medical products, including wound dressings, catheters, implants and medical textiles to prevent infection and potentially could provide alternatives for overcoming the antibiotic resistance [7,8]. Preclinical studies also indicate that AgNPs are cytotoxic to cancers of the brain [9,10], cervix [11], liver [12], colon [13], lung [14], pancreas [15], breast [16], ovary [17], and blood [18,19], and could potentially be used for treatment of cancer. Nevertheless, many questions regarding human health risks still remain. Previous studies have demonstrated associations between AgNPs mediated cytotoxicity, oxidative stress, mitochondrial damage and cell cycle arrest or apoptosis individually in various cell lines [5,20,21,22,23,24]. Additionally, rodent studies have shown dose and size-dependent pulmonary inflammation and altered pulmonary function after inhalation of AgNPs [25,26,27].

Mitochondria are involved in the maintenance of cell viability and function and regulation of cell metabolism. Mitochondrial damage and dysfunction are implicated in aging [28,29], and a range of human diseases, including cancer and neurodegenerative diseases [30,31]. The mitochondria produce ATP through the electron transport chain and are also a major source of reactive oxygen species (ROS) in cells. Low levels of ROS are directly removed by antioxidants within mitochondria; however, during the cellular stress, the accumulation of ROS can damage mitochondrial components, including mitochondrial DNA, protein and lipids. This, in turn, can lead to mitochondrial dysfunction and a further increase in ROS production [32]. Protein cysteine thiols play an important role in mitochondrial antioxidant defense and their modifications are part of redox signal transduction and possibly mitochondria-cell communication [33,34]. The thiols can react with H_2_O_2_ to form highly reactive sulfenic acids [35]. As a first step of many oxidative processes, sulfenic acids are an important target for studying cellular processes involving protein oxidation [36]. Identification of the redox state of mitochondria-specific proteins is challenging. To facilitate studies of mitochondrial protein oxidation, we recently developed a novel probe (3-(2,4-dioxocyclohexyl)propyl-Net_2_-Coumarin (DCP-Net_2_C)) to specifically image sulfenylated proteins in mitochondria [37], which enables examination of the effects of AgNPs on mitochondrial protein oxidation in intact cells.

Because the lung is a likely site for environmental and occupational exposure to AgNPs, in this work, we describe the effects of AgNPs and ionizing radiation on mitochondrial redox state and function in a diverse panel of lung cell lines. In order to understand and potentially mitigate long term adverse health effects that may occur as a result of exposure to AgNPs, especially following inhalation, it is important to examine the sublethal effects of AgNPs on mitochondria. As other ROS inducers, including ionizing radiation, can potentially increase the toxic effects of AgNPs [38,39] we have also investigated the combined effects of AgNPs and radiation on mitochondria. We show that the exposure to AgNPs increases mitochondrial ROS and protein oxidation in a dose- and time-dependent manner impacting cell cycle and cell proliferation. Nevertheless, there was variability in response to AgNPs between the cell lines and the sensitivity to AgNPs was associated with increased mitochondrial spare respiratory capacity and the mitochondrial content in cells. With the exception of NCI-H358, ionizing radiation did not significantly impact the sensitivity to AgNPs.

## 2. Materials and Methods 

### 2.1. Cell Culture 

The experiments were performed with a panel of four lung cell lines: Three cancer cell lines (A549, Calu-1 and NCI-H358) and one transformed lung epithelial cell line (BEAS-2B). All cell lines were obtained from the American Type Culture Collection (ATCC). Cells were cultured at 37 °C under 5% CO_2_, in Ham’s F-12K (Kaighn’s) medium (Gibco/Thermo Fisher Scientific, Waltham, MA, USA) for A549, RPMI-1640 medium (Gibco/Thermo Fisher Scientific, Waltham, MA, USA) for Calu-1 and NCI-H358 and DMEM medium (Gibco/Thermo Fisher Scientific, Waltham, MA, USA) for BEAS-2B. Culture media for all cells was supplemented with 10% fetal bovine serum (Sigma-Aldrich, St. Louis, MO, USA), 100 U/mL penicillin and 100 µg/mL streptomycin (Gibco/Thermo Fisher Scientific, Waltham, MA, USA).

### 2.2. Treatment with AgNPs and Ionizing Radiation

Previous studies indicated that the size and coating of AgNPs could affect the toxicity profile of AgNPs in colorectal cancer and murine monocyte/macrophage cell lines [40]. In contrast, we previously observed that size, shape, and coating of AgNPs did not affect the toxicity profile of AgNPs in triple negative breast cancer cell lines [41]. Thus, the toxicity of AgNPs is determined by both the physical and chemical properties of AgNPs and by the metabolic phenotype of cells. As the goal of the studies reported here was to investigate the cellular determinants of sensitivity to AgNPs, we have used a single type of AgNPs with a minimal polyvinylpyrrolidone coating (PVP, 0.2 wt%; SkySpring Nanomaterials). These AgNPs have been characterized for size, shape, aggregation, hydrodynamic diameter, and ζ-potential in an earlier study [39]. Briefly, transmission electron microscopy (TEM) and dynamic light scattering (DLS) showed that dehydrated AgNPs were generally spherical in shape and 23 ± 14 nm in size. The ζ-potential of AgNPs in the water at pH 7 was approximately −36 mV, indicating good colloidal stability. These particles exist as a heterogeneous mix of different sized aggregates when dispersed in media. For the experiments included here, the cells were exposed to AgNPs in complete medium for the indicated concentrations and durations. 

The ionizing radiation (IR) was applied with a 444 TBq 12,000 Ci self-shielded ^137^Cs (Cesium) irradiator (Mark 1, Model 68A, JL Shepherd and Associates, San Fernando, CA, USA) with indicated doses. The relative timing of AgNPs and IR treatment is described in each figure legend. 

### 2.3. Analysis of Mitochondrial ROS and Protein Oxidation 

For analysis of mitochondrial ROS, the cells were treated with 10 µM MitoPY1 (Tocris Bioscience, Bristol, United Kingdom; mitochondrial H_2_O_2_) or 1 µM MitoSOX (Invitrogen, Carlsbad, CA, USA; mitochondrial superoxide and overall redox state) and analyzed by flow cytometry. Changes in mitochondrial protein oxidation with AgNPs and IR exposure were similarly quantified by flow cytometry after treating the cells with 50 µM mitochondria-targeted probe DCP-Net_2_C [37]. Cell mitochondrial content was quantified with 100 nM MitoTracker Green (Invitrogen, Carlsbad, CA, USA). All cell treatments were for 30 min at 37 °C under 5% CO_2_. 

### 2.4. Transmission Electron Microscopy (TEM)

Cells were treated with AgNPs (10 µg/mL) for 1 h then were washed thoroughly in PBS to remove AgNPs not bound or internalized by cells. Cells were fixed in 2.5% glutaraldehyde at 4 °C overnight. Next, fixed cells were scraped from the wells, pelleted, embedded in resin, cut into ultrathin sections (80 nm) and placed on copper coated formvar grids. All samples were imaged using an FEI Tecnai Spirit transmission electron microscope (Thermo Fisher Scientific, Waltham, MA, USA). Samples were imaged without additional staining to facilitate the detection of AgNPs.

### 2.5. Flow Cytometry (Mitochondrial Content, Mitochondrial Protein Oxidation, Mitochondrial ROS and Cell Cycle Analysis)

After treatment, the cells were washed twice with Dulbecco’s phosphate-buffered saline (DPBS) (Lonza, Basel, Switzerland) and detached from the plates with trypsin. For mitochondrial content and ROS analysis, the cells were resuspended live and kept on ice. For the analysis of mitochondrial protein oxidation and cell cycle, the cells were fixed for 5 min with cold (−20 °C) methanol or for 30 min with 70% ethanol, respectively. Fixed cells were washed with DPBS before resuspending for analysis. For the analysis of the cell cycle, cells were treated with 100 µg/mL RNAse (Invitrogen, Carlsbad, USA) and stained with 50 µg/mL propidium iodide (Invitrogen, Carlsbad, CA, USA). The flow cytometry experiments were conducted with a BD FACS Canto II Cell Analyzer (BD Biosciences, San Jose, CA, USA), and 10,000–50,000 cells were analyzed for each condition. Each experiment was repeated at least three times independently. Data were analyzed using the FCS Express 6 Flow software (De Novo Software, Pasadena, CA, USA), and the Students’ *t*-test was used to compare the mean fluorescence values between different conditions. 

### 2.6. Cell Proliferation and ATP Assay

The cell proliferation was analyzed with CyQUANT assay (Thermo Fisher Scientific, Waltham, MA, USA) according to the manufacturer’s protocol, and the fluorescence was read with a Spark microplate reader (Tecan, Männedorf, Switzerland). The ATP content in cells was measured with CellTiter-Glo 3D assay (Promega, Madison, WI, USA) according to the manufacturer’s protocol, and the luminescence was read with the Spark microplate reader. All conditions were measured in three technical replicates, and each experiment was repeated independently three times. The Students’ *t*-test was used to compare the mean values between different conditions.

### 2.7. Mitochondrial Respirometry Analysis

The effects of AgNPs on the mitochondrial function were measured using the Seahorse Mito Stress Test following the manufacturer’s protocol. Briefly, the day before the AgNP treatment, 1.5 × 10^4^ cells/well were plated on a Seahorse plate. The next day 10 µg/mL AgNPs were added to the assigned wells and incubated for 24 h. On the day of the experiment, the assay media, compounds (to final concentrations of 1 μM for Oligomycin (Thermo Fisher Scientific, Waltham, MA, USA), 1 μM for Carbonyl cyanide 4-(trifluoromethoxy) phenylhydrazone (FCCP) (Cayman Chemical, Ann Arbor, MI, USA) and 1 μM Antimycin A (Abcam, Cambridge, United Kingdom)/Rotenone (Sigma-Aldrich, St. Louis, MO, USA) and cells were prepared according to Seahorse protocols. The experiment was run with Seahorse XF24 (Agilent Technologies, Santa Clara, CA, USA), following injection order: (1) AgNPs or vehicle media (1 h reading); (2) Oligomycin; (3) FCCP; and (4) Antimycin A/Rotenone. After the Seahorse analysis, the cells were lysed with modified RIPA buffer (50 mM Tris-HCl, pH 7.4; 1% NP40; 0.25% Sodium deoxycholate; 15 mM NaCl; 1 mM EDTA; 1 mM NaF; supplemented with Roche (Basel, Switzerland) protease and phosphatase inhibitor tablets) and protein concentration was measured with bicinchoninic acid assay (Thermo Fisher Scientific, Waltham, MA, USA) for data normalization. Each experiment was repeated at least two times independently. The data were analyzed with Wave software (Agilent Technologies, Santa Clara, CA, USA), and the Students’ t-test was used to compare the mean fluorescence values between different conditions.

## 3. Results

### 3.1. Cell Proliferation

**Figure 1:**
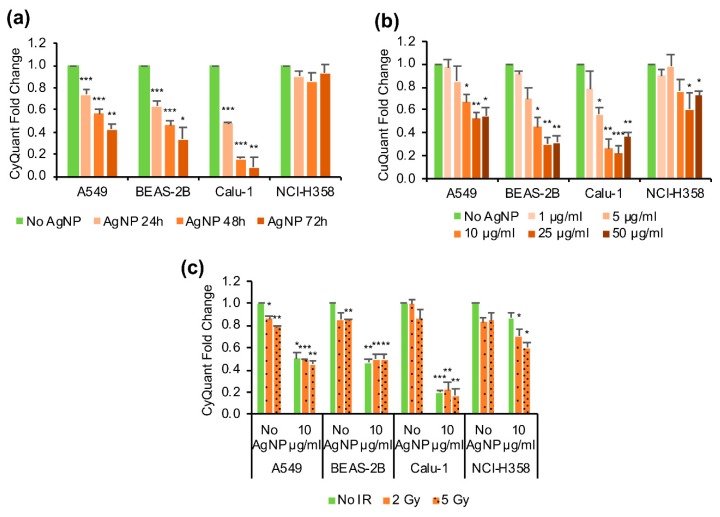
Effect of AgNP exposure and ionizing radiation on cell proliferation. (**a**) Effect of AgNP exposure time. The cells were treated with 10 μg/mL AgNPs for 24 h, 48 h and 72 h, as indicated. (**b**) Effect of AgNP concentration. The cells were treated for 48 h with different doses of AgNPs as indicated. (**c**) Effect of AgNP exposure combined with ionizing radiation (IR). The cells were treated with 10 μg/mL AgNPs and 2 Gy or 5 Gy ionizing radiation (IR) immediately after the start of the AgNP exposure, and the cell proliferation was measured after 48 h. Data are presented as mean fold change relative to the untreated conditions, the error bars represent the standard error of the mean. * *p* = 0.01–0.05, ** *p* = 0.001–0.01, *** *p* < 0.001, calculated relative to the untreated conditions using Students’ *t*-test.

Changes in cell proliferation were measured using CyQuant proliferation-assay. The response to the exposure to AgNPs was both time- (Figure 1a) and dose-dependent (Figure 1b), but the response varied between the cell lines. The most sensitive cell line to the AgNP treatment was Calu-1, followed by BEAS-2B and A549. NCI-H358 was the least sensitive (most tolerant). Additional treatment with ionizing radiation using either 2 Gy or 5 Gy did not further decrease the cell proliferation compared to the AgNP treatment only, except for the NCI-H358 cell line that otherwise was comparatively tolerant to both AgNP treatment and ionizing radiation (*p* = 0.106 for AgNP with 2 Gy irradiation and *p* = 0.014 for AgNP with 5 Gy irradiation as compared with AgNP only).

**Figure 2:**
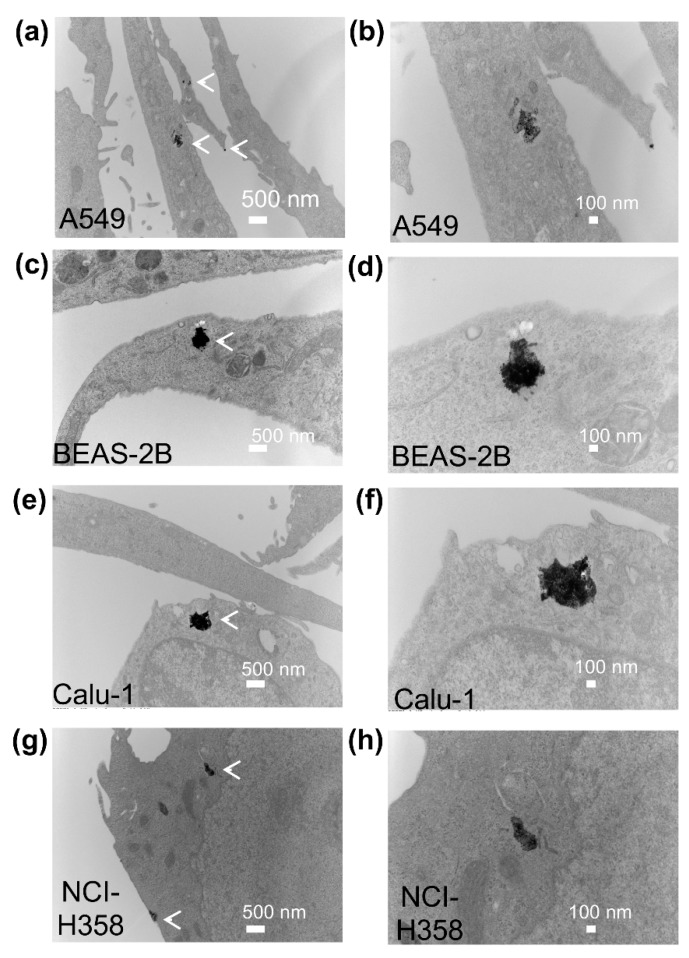
Uptake and localization of AgNPs in lung cell lines. The subcellular localization of AgNPs in A549 (**a**,**b**), BEAS-2B (**c**,**d**), Calu-1 (**e**,**f**), and NCI-H358 cells (**g**,**h**) was imaged by transmission electron microscopy 1 h after cells were exposed to AgNPs (10 μg/mL). Representative images are shown. Magnification was 11,000× for (**a**,**c**,**e**,**g**). Additional images of the areas shown in the left panels are included at higher magnification (30,000×) in the panels on the right (**b**,**d**,**f**,**h**). Clusters of AgNPs in vesicles are identified by white arrows in the lower magnification images.

To determine if the varied sensitivity of the four cell lines to AgNPs was due to differential uptake of AgNPs into cells, we examined the uptake and subcellular localization of AgNPs using transmission electron microscopy (TEM). AgNPs were internalized within 1 h and were found in membrane bound vesicles, likely endosomes, in all cell lines after 1 h exposure to AgNPs (Figure 2). The cell lines most sensitive to AgNPs (Calu-1 and BEAS-2B) appear to have higher intracellular levels of AgNPs compared with A549 and NCI-H358 cells.

### 3.2. Cell Cycle

**Figure 3:**
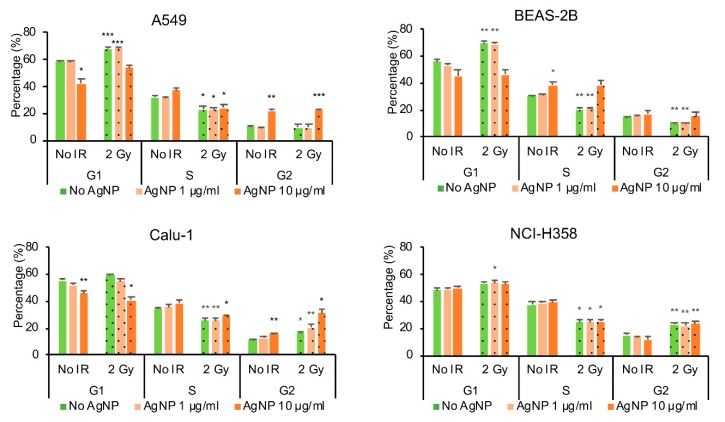
Effect of AgNP exposure and ionizing radiation (IR) on the cell cycle The cells were treated with 10 μg/mL AgNPs for 24 h and 2 Gy IR immediately after the start of the AgNP exposure, stained with PI and analyzed by flow cytometry 24 h after AgNP exposure and IR. Data are presented as mean value, and the error bars represent the standard error of the mean. * *p* = 0.01–0.05, ** *p* = 0.001–0.01, *** *p* < 0.001, calculated relative to the untreated conditions using Students’ *t*-test.

The cell cycle was studied with flow cytometry using PI-staining. Exposure to AgNPs and ionizing radiation affected the cell cycle in all the cell lines studied (Figure 3), but the changes were cell line specific. The ionizing radiation-induced G1 phase arrest in the cells that contained the wild-type *TP53* (A549 and BEAS-2B), whereas in the *TP53* null cell lines (Calu-1 and NCI-H358) the cells accumulated mainly in the G2 phase. The population of cells in S-phase decreased after ionizing radiation in all cell lines. On the other hand, the AgNP exposure induced G2 arrest in A549 and Calu-1 cell lines, S-phase arrest in BEAS-2B and did not seem to have any effect on the cell cycle in the NCI-H358 cells. Only in the Calu-1 cells the exposure to combined AgNPs and ionizing radiation appeared to have a statistically significant effect on the increase in cell accumulation in G2 phase (*p* = 0.037 for 1 μg/mL AgNPs with 2 Gy irradiation and *p* = 0.028 for 10 μg/mL AgNPs with 2 Gy irradiation as compared with AgNPs only).

### 3.3. Mitochondrial Reactive Oxygen Species (ROS)

**Figure 4:**
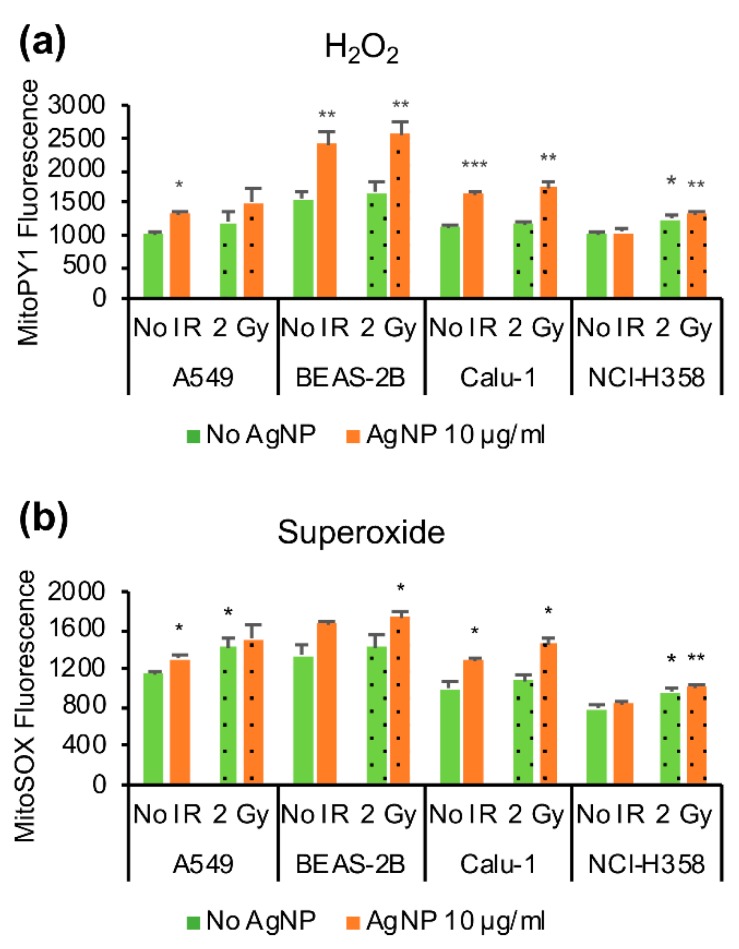
Effect of AgNP exposure and ionizing radiation (IR) on mitochondrial ROS. (**a**) Mitochondrial H_2_O_2_. The cells were treated with 10 μg/mL AgNPs for 24 h and 2 Gy IR immediately after the start of AgNP exposure, stained with MitoPY1 and analyzed by flow cytometry. (**b**) Mitochondrial superoxide. The cells were treated with 10 μg/mL AgNPs for 24 h and 2 Gy IR immediately after the start of the AgNP exposure, stained with MitoSOX, and analyzed by flow cytometry. Data are presented as mean fluorescence value, the error bars represent the standard error of the mean. * *p* = 0.01–0.05, ** *p* = 0.001–0.01, *** *p* < 0.001, calculated relative to the untreated conditions using Students’ *t*-test.

The mitochondrial ROS was measured in live cells using flow cytometry. MitoPY1-probe was used for H_2_O_2_ detection and MitoSOX as a more general sensor of superoxide and mitochondrial redox state. The cell lines in our study had different basal levels of mitochondrial superoxide whereas the mitochondrial H_2_O_2_ levels were more similar, except for BEAS-2B that had the highest basal level of both H_2_O_2_ and superoxide (Figure 4). The cell line most resistant to AgNPs, NCI-H358, displayed the lowest level of mitochondrial superoxide (*p* = 0.001 compared with A549, *p* = 0.046 compared with BEAS-2B and *p* = 0.032 compared with Calu-1). Exposure to AgNPs increased both mitochondrial H_2_O_2_ (Figure 4a) and mitochondrial superoxide (Figure 4b) in all cell lines except in the resistant NCI-H358. On the other hand, the ionizing radiation increased both mitochondrial H_2_O_2_ and superoxide in the NCI-H358; whereas, the effect of ionizing radiation was much smaller in other cell lines and not statistically significant. Also, there appeared to be a small increase in mitochondrial ROS with combined exposure of AgNPs and ionizing radiation, but this was statistically significant only for superoxide in Calu-1 cells (*p* = 0.02 as compared to cells treated only with AgNPs).

### 3.4. Mitochondrial Protein Oxidation

**Figure 5:**
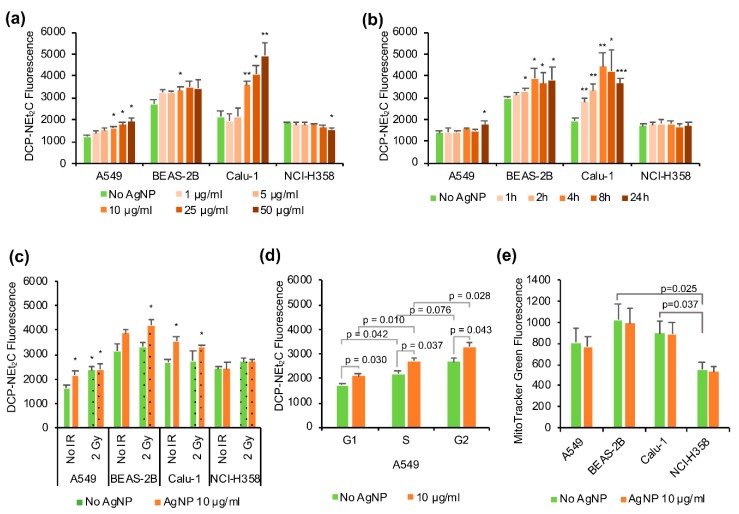
Mitochondrial protein oxidation and mitochondrial content. (**a**) Changes in mitochondrial protein oxidation with different AgNP exposure times. The cells were treated with 10 μg/mL AgNPs for different time durations as indicated, stained with DCP-NEt_2_C and analyzed by flow cytometry. (**b**) Changes in mitochondrial protein oxidation with different doses of AgNPs. The cells were treated for 24 h with different doses of AgNPs as indicated, stained with DCP-NEt_2_C and analyzed by flow cytometry. (**c**) Effect of AgNPs exposure combined with ionizing radiation (IR). The cells were treated with 10 μg/mL AgNPs for 24 h and 2 Gy IR immediately after the start of the AgNP exposure, stained with DCP-NEt_2_C and analyzed by flow cytometry. (**d**) Mitochondrial protein oxidation in different cell cycle phases. The A549 cells were treated with 10 µg/mL AgNPs for 24 h, stained with DCP-NEt_2_C and PI and analyzed by flow cytometry. (**e**) Mitochondrial content in different cell lines was measured with MitoTracker Green. Cells were treated with 10 μg/mL AgNP for 24 h, stained with MitoTracker Green and analyzed by flow cytometry. Data are presented as mean relative fluorescence, the error bars represent the standard error of the mean. * *p* = 0.01–0.05, ** *p* = 0.001–0.01, *** *p* < 0.001, calculated relative to the untreated conditions using Students’ *t*-test.

Mitochondrial protein oxidation was analyzed using the recently developed probe DCP-NEt_2_C containing a protein sulfenic acid trapping moiety for detecting oxidized proteins and a coumarin moiety for the localization into the mitochondria and fluorescence detection [37]. In line with the mitochondrial ROS profiles, the AgNP exposure increased mitochondrial protein oxidation in a time and dose-dependent manner matching the sensitivity to AgNPs with the highest oxidation noted for Calu-1, followed by BEAS-2B, A549, and lastly NCI-H358 cells. The cell lines most sensitive to AgNPs (Calu-1 and BEAS-2B) showed higher level of basal mitochondrial protein oxidation (e.g., BEAS-2B relative to A549 and NCI-H358) or rapid increase in mitochondrial protein oxidation with the exposure time (Calu-1), whereas in A549 and NCI-H358 the protein oxidation increased only at the later time points or not at all (Figure 5a).

Mitochondrial protein oxidation also increased in the AgNP-sensitive cell lines as a function of increasing concentrations of AgNPs, and the increased oxidation was statistically significant in particular for concentrations greater than 10 μg/mL (Figure 5b). The 2 Gy radiation increased mitochondrial protein oxidation significantly only in A549 cells and did not seem to have synergistic effects with AgNP exposure on mitochondrial protein oxidation (Figure 5c).

To verify that the changes in the level of protein oxidation labeling were not due to changes in mitochondrial content induced by treatment with AgNPs, we used MitoTracker Green to quantify mitochondrial content in the cell. We did not detect any significant change with AgNP treatment in the four cell lines studied here (Figure 5e). However, the cell lines most sensitive to AgNPs (Calu-1 and BEAS-2B) showed higher mitochondrial content under basal conditions compared with NCI-H358 cells (Figure 5e). Additionally, different cell cycle phases have been reported to have different amounts of mitochondria and mitochondrial activity [42]. Therefore, we also analyzed the data stratified by cell cycle phase to ensure that the changes observed in the mitochondrial protein oxidation are not artifacts associated with differences in cell cycle distribution. As shown in Figure 5d for A549 cells as an example, the G2 phase has the highest amount of DCP-NEt_2_C labeling, followed by S-phase and G1 having the lowest amount of labeling. Nevertheless, the changes induced by the AgNP exposure on mitochondrial protein oxidation could be seen in all cell cycle phases and are, thus, not linked to the mitochondrial content of cell cycle phases. The same results were obtained for the other three cell lines (data not shown).

### 3.5. Mitochondrial Function

**Figure 6:**
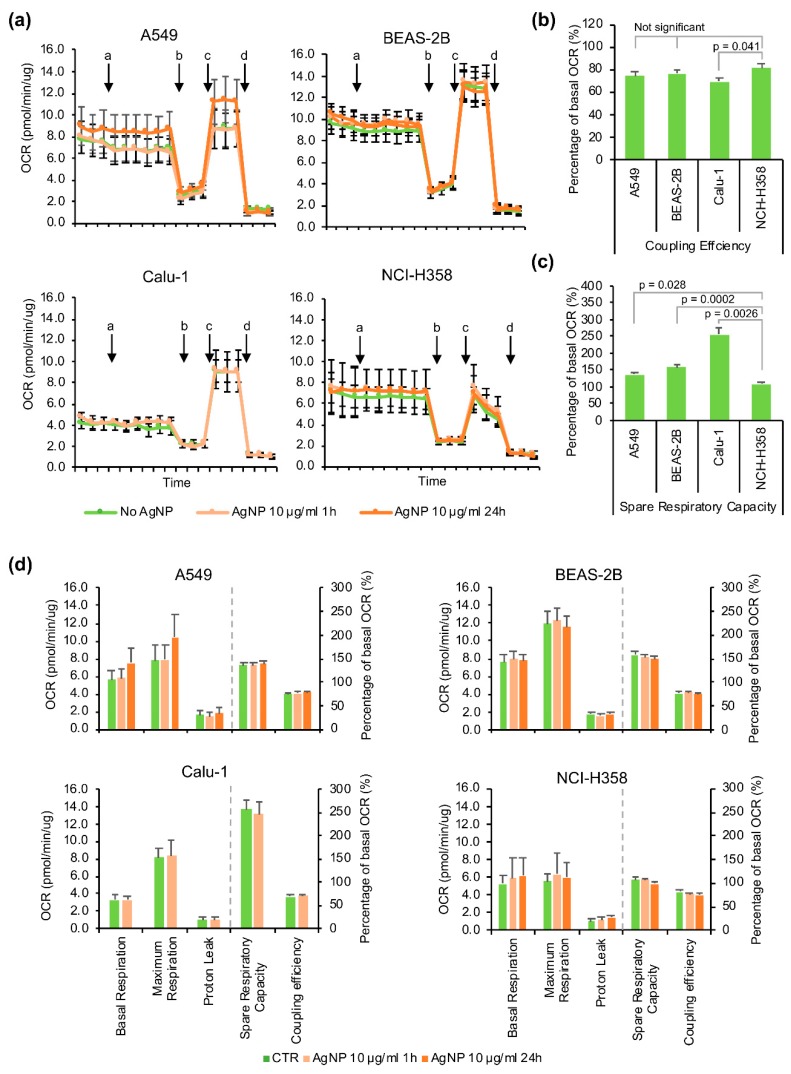
Effects of AgNPs exposure on the mitochondrial respiration. (**a**) Oxygen consumption rate (OCR) measured by Seahorse MitoStress assay. a: addition of 10 μg/mL AgNPs for 1 h exposure or vehicle media for the untreated controls and samples pre-treated with 10 μg/mL AgNP for 23 h, b: oligomycin, c: FCCP, d: Antimycin A/Rotenone. (**b**) Comparison of spare respiratory capacity in different cell lines. (**c**) Comparison of coupling efficiency in different cell lines. (**d**) Quantification of key parameters of mitochondrial function. Data are presented as mean value, the error bars represent the standard error of the mean. Statistical values were calculated using Students’ *t*-test.

The mitochondrial respiration was evaluated with the Seahorse MitoStress assay (Figure 6) after 1 h or 24 h exposure to 10 μg/mL AgNPs. In the case of Calu-1 cells, only the 1 h exposure was analyzed as these cells showed a high degree of cell death at 24 h.

Overall, while there appeared to be some increase in the basal respiration, proton leak and decrease in spare respiratory capacity and coupling efficiency with the AgNP treatment, these changes were not statistically significant (Figure 6d). However, the AgNP sensitive cell lines BEAS-2B and Calu-1 had the highest spare respiratory capacity in the untreated conditions, followed by A549 and lastly the most resistant (NCI-H358), which had almost no spare respiratory capacity (Figure 6b). The sensitive cells BEAS-2B and Calu-1 also had about twice as much mitochondrial content compared to NCI-H358 as measured with MitoTracker Green (Figure 5e), which could partly explain the higher spare respiratory capacity. Among the four cell lines, the Calu-1 had the lowest mitochondrial coupling efficiency (Figure 6c).

**Figure 7:**
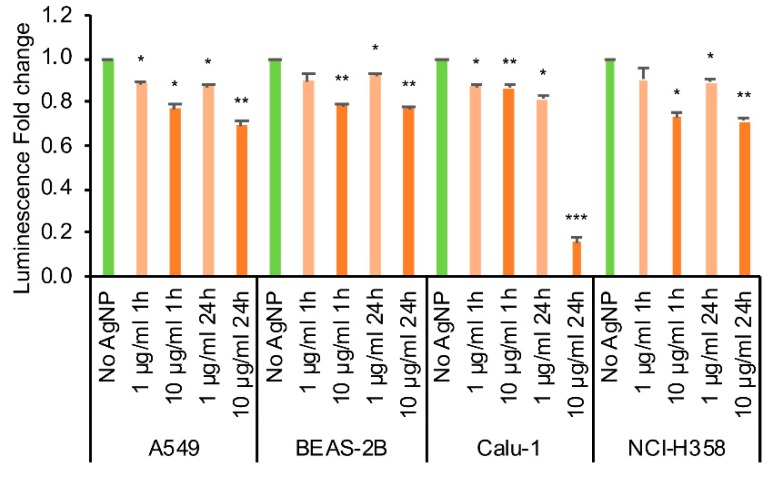
ATP content in cells. The cells were treated with 1 or 10 μg/mL AgNP for 1 h or 24 h and the ATP content was measured using CellTiter Glo assay. * *p* = 0.01–0.05, ** *p* = 0.001–0.01, *** *p* < 0.001 calculated relative to the untreated conditions using Students’ *t*-test.

Thus, despite the changes in mitochondrial ROS and protein oxidation induced by AgNP treatment, mitochondrial respiration and ATP production/coupling efficiency were not impaired by treatment. In order to account for non-mitochondrial effects on ATP levels, we also measured the ATP content in the cells using Promega CellTiter Glo-assay (Figure 7). Treatment with AgNPs decreased ATP content in cells within one hour for both 1 μg/mL, and 10 μg/mL concentrations and further exposure for 24 h only decreased ATP slightly, except for Calu-1 cells where the cell viability was almost completely lost at 24 h. For the other cell lines, the cells exposed to 10 μg/mL AgNPs for 24 h had statistically significantly less ATP than the cells exposed to 1 μg/mL AgNPs (A549: *p* = 0.019, BEAS-2B: *p* = 0.0.10, NCI-H358: *p* = 0.011).

## 4. Discussion

Several recent studies have shown that exposure to AgNPs is cytotoxic in different cell lines, but with variable response [1,39,40,43]. In addition to the cell type, the particle size, type, surface coating and exposure time also play a role in the cellular toxicity [39,40]. In general, it is thought that AgNPs enter the cell by phagocytosis or endocytosis and once inside the cell AgNPs, or ionized Ag^+^ released from the nanoparticles, induce ROS and disrupt mitochondrial function [1,5,25,44]. Mitochondria play key roles in many cell processes, including energy production, regulation of cell metabolism and cell viability, synthesis of heme and iron-sulfur clusters, and regulation of calcium, copper, manganese and iron levels. The mitochondrial respiratory chain is one of the major sources of ROS in the cell, which under normal conditions are kept in balance by mitochondrial antioxidant systems. During cellular stress, the mitochondria may become dysfunctional, which can result in increased ROS production and cause cellular damage and cell death [36,45].

The studies presented here were designed to mimic potential environmental exposure to AgNPs. In this case, both intracellular and extracellular exposure to AgNPs can have effects. Thus, all cell lines were exposed to equivalent doses of AgNPs, in equivalent volumes of culture media, and cells were plated in identically sized wells at similar cell density, ensuring comparable exposure to AgNPs. Furthermore, the data were collected at multiple time points and doses of AgNPs to be able to assess differences in the timing, magnitude, and type of responses detected among cell types.

We initially evaluated the AgNP-induced cell toxicity using a battery of tests starting with the effects on cell proliferation. As others have reported in various cell lines [1,5,15,43], we found that the decrease in proliferation with exposure to AgNPs was both time- and dose-dependent. In our panel of four lung cell lines, the most sensitive to the AgNPs was the Calu-1 cell line, followed by BEAS-2B and A549. The most resistant cell line to AgNP exposure was NCI-H358, where decrease in proliferation was seen only at higher concentrations of AgNPs or when combined with ionizing radiation. In the other cell lines, additional irradiation did not further decrease cell proliferation as compared to the AgNP treatment alone. Earlier studies have reported radiosensitizing properties for AgNPs [9,46,47,48], but our results demonstrate that this might be dependent on the cell type and dose of both IR and AgNPs.

AgNPs have been shown to induce DNA damage, cell death by apoptosis and necrosis, and also cell cycle arrest in different cell lines [1]. The effects on the cell cycle seem to be cell type-dependent; in some studies, S-phase arrest has been reported, whereas others point to G2-phase arrest [1,23,44,49,50,51,52]. In our study, A549 and Calu-1 cells showed accumulation in the G2-phase after AgNP exposure, whereas the BEAS-2B cells accumulated in the S-phase. A549 and Calu-1 also seemed to have some accumulation in the S-phase, but this was not statistically significant. The NCI-H358 cells did not show cell cycle changes related to AgNP exposure. The ionizing radiation caused cell accumulation in the G1-phase in A549 and BEAS-2B cells containing the wild type *TP53*, and decrease of cells in the S-phase in all cell lines. Interestingly, the effects of ionizing radiation on cell cycle seemed to be different from the AgNP exposure, and there were no combined effects, even though in addition to DNA damage, ionizing radiation can also induce mitochondrial dysfunction and increase in cellular and mitochondrial ROS [53,54,55].

Since the mitochondria appear to be an important target for the effects of AgNPs, we wanted to analyze the redox state of mitochondria by studying both the mitochondrial ROS and protein oxidation. We used two mitochondrial fluorescent probes to study ROS, MitoPY1 for H_2_O_2_ and MitoSOX as an indicator of superoxide and other ROS. Both of them showed an increase in fluorescence after 24 h exposure to AgNPs in the AgNP-responsive cell lines (A549, BEAS-2B and Calu-1), but not in the AgNP-tolerant NCI-H358 cell line. In line with these results, there was also a time- and dose-dependent increase in the mitochondrial protein oxidation in the AgNP-sensitive cell lines, but not in the NCI-H358 cells, where a slight decrease was observed at higher concentrations of AgNPs. The TEM imaging showed internalized AgNPs in all cell lines after 1 h exposure, but at higher level in the AgNP-sensitive BEAS-2B and Calu-1 cells, correlating with the stronger increase in protein oxidation (occurring at earlier time points and at lower doses of AgNPs) compared with A549 and NCI-H358 cells. Interestingly, ionizing radiation increased mitochondrial superoxide and protein oxidation only in the A549 cells, which was not further increased with exposure to AgNPs. The different phases of the cell cycle have different redox states [42,56], and a redox control of the cell cycle has been proposed [57]. In our study, changes in cell cycle distribution with AgNP treatment were only seen in the cell lines that also had increased mitochondrial ROS and protein oxidation. Of note, even though the NCI-H358 cells showed decreased cell proliferation for combined AgNPs and ionizing radiation treatment, this was not reflected in the mitochondrial ROS or protein oxidation.

We also analyzed the effects of AgNPs on mitochondrial respiration using Seahorse MitoStress assay. The AgNP-responsive cell lines had higher spare respiratory capacity both in the absence and presence of AgNPs, possible due to higher mitochondrial content, than the tolerant NCI-H358. Interestingly, Calu-1, the cell line most sensitive to AgNPs had also the lower capacity for ATP-production. The mitochondrial spare respiratory capacity seemed to decrease slightly with the AgNP treatment along with an increase in the proton leak pointing to mitochondrial dysfunction, whereas the basal respiration appeared to increase. In our study, the changes in the basal respiration, spare respiratory capacity, coupling efficiency or other key parameters of mitochondria function induced by AgNPs were not statistically significant in any of the four cell lines investigated. The effects of AgNPs on mitochondrial respiration have been little studied, but there has been at least one report also showing increased oxygen consumption after exposure to AgNPs in murine hippocampal neuronal HT22 cells [58].

However, measurement of cellular ATP showed a dose-dependent decrease in ATP levels as early as 1 h of AgNP exposure. Based on TEM analysis, at this time point the AgNPs were primarily localized to endosomal vesicles suggesting the effects were likely caused by the release of Ag^+^ rather than direct interaction of AgNPs with mitochondria. There was only a small additional decrease in ATP with longer exposure to AgNPs, except for Calu-1 which showed almost complete loss of ATP and cell viability after 24 h. Interestingly, a similar decrease in ATP was observed in NCI-H358 cells and yet this did not impact its cellular proliferation. Other studies have also shown a decrease in ATP levels after exposure to AgNPs and loss of mitochondrial membrane potential (reviewed in Reference [44,59]). It is possible that the increase in the mitochondrial respiration is a response to lowering levels of ATP in the cells. This increase, in turn, could lead to more mitochondrial ROS, especially in compromised mitochondria.

While the clinical significance of our findings remains to be established, the data are consistent with the pathology of exposure to AgNPs. AgNPs are known to penetrate the lung structures deep into the alveoli through diffusion and to cross the blood-air barrier of the lungs leading to lung inflammation and fibrosis, as well as toxicity to other organs (e.g., kidney, liver) [60]. Interestingly, Calu-1 and NCI-H358 situated at the extremes of sensitivity to AgNPs in our panel of cell lines, both have epithelial phenotype, but were isolated from different anatomical sites. AgNPs resistant NCI-H358 cells have bronchoalveolar origin, while AgNPs sensitive Calu-1 cells were isolated from the lung pleura suggesting the cells lining the lungs may have increased sensitivity to AgNPs. Supporting this hypothesis, published in vivo data showed that systemic administration of silver nitrate induces pleural inflammation and fibrosis in rabbits [61], and ex vivo studies showed that exposure of sheep pleura to AgNPs increases its permeability [62]. Future in vivo investigations will address the dependence of sensitivity to AgNPs on mitochondrial mass and redox state across the anatomy of lung.

## 5. Conclusions

The results of the studies presented here reveal a broad variability in the sensitivity of lung cells to AgNPs determined by several contributing processes: cellular uptake of AgNPs and conversion to Ag^+^, intracellular relay of redox signaling from endosomes to mitochondria, and the intrinsic capacity of individual cell lines to interfere with this relay and/or respond to mitochondrial oxidative challenge. We have shown that exposure to AgNPs decreases cell proliferation and induces cell cycle arrest in A549, BEAS-2B and Calu-2, but not in NCI-H358 cells. The AgNPs increase mitochondrial ROS and protein oxidation in time- and dose-dependent manner in the sensitive cell lines, but without altering the mitochondrial respiration significantly. The change in mitochondrial redox state was accompanied by a decrease in cellular ATP levels, including in the NCI-H358 cells resistant to AgNPs treatment. The more sensitive cell lines had more mitochondrial content and spare respiratory capacity, potentially making them more susceptible to mitochondrial damage leading to increased ROS levels. Interestingly, ionizing radiation seemed to have separate effects from AgNP exposure, and there was no interaction with AgNPs in most cell lines with the exception of NCI-H358 which showed decreased proliferation with combined radiation and AgNPs treatment at higher doses of radiation.

## References

[1] Akter M., Sikder M.T., Rahman M.M., Ullah A., Hossain K.F.B., Banik S., Hosokawa T., Saito T., Kurasaki M. (2018). A systematic review on silver nanoparticles-induced cytotoxicity: Physicochemical properties and perspectives. J. Adv. Res..

[2] Nymark P., Kohonen P., Hongisto V., Grafstrom R.C. (2018). Toxic and Genomic Influences of Inhaled Nanomaterials as a Basis for Predicting Adverse Outcome. Ann. Am. Thorac. Soc..

[3] Shi J.J., Kantoff P.W., Wooster R., Farokhzad O.C. (2017). Cancer nanomedicine: Progress, challenges and opportunities. Nat. Rev. Cancer.

[4] Burdusel A.C., Gherasim O., Grumezescu A.M., Mogoanta L., Ficai A., Andronescu E. (2018). Biomedical Applications of Silver Nanoparticles: An Up-to-Date Overview. Nanomaterials.

[5] Jeong J.K., Gurunathan S., Kang M.H., Han J.W., Das J., Choi Y.J., Kwon D.N., Cho S.G., Park C., Seo H.G. (2016). Hypoxia-mediated autophagic flux inhibits silver nanoparticle-triggered apoptosis in human lung cancer cells. Sci. Rep..

[6] McGillicuddy E., Murray I., Kavanagh S., Morrison L., Fogarty A., Cormican M., Dockery P., Prendergast M., Rowan N., Morris D. (2017). Silver nanoparticles in the environment: Sources, detection and ecotoxicology. Sci. Total Environ..

[7] Chen X., Schluesener H.J. (2008). Nanosilver: A nanoproduct in medical application. Toxicol. Lett..

[8] Dos Santos C.A., Seckler M.M., Ingle A.P., Gupta I., Galdiero S., Galdiero M., Gade A., Rai M. (2014). Silver nanoparticles: Therapeutical uses, toxicity, and safety issues. J. Pharm. Sci..

[9] Liu P.D., Huang Z.H., Chen Z.W., Xu R.Z., Wu H., Zang F.C., Wang C.L., Gu N. (2013). Silver nanoparticles: A novel radiation sensitizer for glioma?. Nanoscale.

[10] Locatelli E., Naddaka M., Uboldi C., Loudos G., Fragogeorgi E., Molinari V., Pucci A., Tsotakos T., Psimadas D., Ponti J. (2014). Targeted delivery of silver nanoparticles and alisertib: In vitro and in vivo synergistic effect against glioblastoma. Nanomedicine.

[11] Miura N., Shinohara Y. (2009). Cytotoxic effect and apoptosis induction by silver nanoparticles in HeLa cells. Biochem. Biophys. Res. Commun..

[12] Kawata K., Osawa M., Okabe S. (2009). In vitro toxicity of silver nanoparticles at noncytotoxic doses to HepG2 human hepatoma cells. Environ. Sci. Technol..

[13] Sanpui P., Chattopadhyay A., Ghosh S.S. (2011). Induction of Apoptosis in Cancer Cells at Low Silver Nanoparticle Concentrations using Chitosan Nanocarrier. Acs Appl. Mater. Interfaces.

[14] Beer C., Foldbjerg R., Hayashi Y., Sutherland D.S., Autrup H. (2012). Toxicity of silver nanoparticles-Nanoparticle or silver ion?. Toxicol. Lett..

[15] Zielinska E., Zauszkiewicz-Pawlak A., Wojcik M., Inkielewicz-Stepniak I. (2018). Silver nanoparticles of different sizes induce a mixed type of programmed cell death in human pancreatic ductal adenocarcinoma. Oncotarget.

[16] Liu J.H., Zhao Y.X., Guo Q.Q., Wang Z., Wang H.Y., Yang Y.X., Huang Y.Z. (2012). TAT-modified nanosilver for combating multidrug-resistant cancer. Biomaterials.

[17] Fahrenholtz C.D., Swanner J., Ramirez-Perez M., Singh R.N. (2017). Heterogeneous Responses of Ovarian Cancer Cells to Silver Nanoparticles as a Single Agent and in Combination with Cisplatin. J. Nanomater..

[18] Guo D., Zhu L., Huang Z., Zhou H., Ge Y., Ma W., Wu J., Zhang X., Zhou X., Zhang Y. (2013). Anti-leukemia activity of PVP-coated silver nanoparticles via generation of reactive oxygen species and release of silver ions. Biomaterials.

[19] Guo D.W., Zhao Y., Zhang Y., Wang Q., Huang Z.H., Ding Q., Guo Z.R., Zhou X.F., Zhu L.Y., Gu N. (2014). The Cellular Uptake and Cytotoxic Effect of Silver Nanoparticles on Chronic Myeloid Leukemia Cells. J. Biomed. Nanotechnol..

[20] Carlson C., Hussain S.M., Schrand A.M., Braydich-Stolle L.K., Hess K.L., Jones R.L., Schlager J.J. (2008). Unique cellular interaction of silver nanoparticles: Size-dependent generation of reactive oxygen species. J. Phys. Chem. B.

[21] Foldbjerg R., Dang D.A., Autrup H. (2011). Cytotoxicity and genotoxicity of silver nanoparticles in the human lung cancer cell line, A549. Arch. Toxicol..

[22] Lee Y.H., Cheng F.Y., Chiu H.W., Tsai J.C., Fang C.Y., Chen C.W., Wang Y.J. (2014). Cytotoxicity, oxidative stress, apoptosis and the autophagic effects of silver nanoparticles in mouse embryonic fibroblasts. Biomaterials.

[23] AshaRani P.V., Low Kah Mun G., Hande M.P., Valiyaveettil S. (2009). Cytotoxicity and genotoxicity of silver nanoparticles in human cells. ACS Nano.

[24] Zhu L., Guo D., Sun L., Huang Z., Zhang X., Ma W., Wu J., Xiao L., Zhao Y., Gu N. (2017). Activation of autophagy by elevated reactive oxygen species rather than released silver ions promotes cytotoxicity of polyvinylpyrrolidone-coated silver nanoparticles in hematopoietic cells. Nanoscale.

[25] Guo C., Buckley A., Marczylo T., Seiffert J., Romer I., Warren J., Hodgson A., Chung K.F., Gant T.W., Smith R. (2018). The small airway epithelium as a target for the adverse pulmonary effects of silver nanoparticle inhalation. Nanotoxicology.

[26] Braakhuis H.M., Gosens I., Krystek P., Boere J.A., Cassee F.R., Fokkens P.H., Post J.A., van Loveren H., Park M.V. (2014). Particle size dependent deposition and pulmonary inflammation after short-term inhalation of silver nanoparticles. Part Fibre Toxicol..

[27] Seiffert J., Buckley A., Leo B., Martin N.G., Zhu J., Dai R., Hussain F., Guo C., Warren J., Hodgson A. (2016). Pulmonary effects of inhalation of spark-generated silver nanoparticles in Brown-Norway and Sprague-Dawley rats. Respir. Res..

[28] Tyrrell D.J., Bharadwaj M.S., Van Horn C.G., Kritchevsky S.B., Nicklas B.J., Molina A.J. (2015). Respirometric Profiling of Muscle Mitochondria and Blood Cells Are Associated With Differences in Gait Speed Among Community-Dwelling Older Adults. J. Gerontol. A Biol. Sci. Med. Sci..

[29] Bharadwaj M.S., Tyrrell D.J., Leng I., Demons J.L., Lyles M.F., Carr J.J., Nicklas B.J., Molina A.J. (2015). Relationships between mitochondrial content and bioenergetics with obesity, body composition and fat distribution in healthy older adults. BMC Obes..

[30] Wallace D.C. (2012). Mitochondria and cancer. Nat. Rev.Cancer.

[31] Arun S., Liu L., Donmez G. (2016). Mitochondrial Biology and Neurological Diseases. Curr. Neuropharmacol..

[32] Saito T., Sadoshima J. (2015). Molecular mechanisms of mitochondrial autophagy/mitophagy in the heart. Circ. Res..

[33] Requejo R., Chouchani E.T., Hurd T.R., Menger K.E., Hampton M.B., Murphy M.P. (2010). Measuring mitochondrial protein thiol redox state. Methods Enzymol..

[34] Caito S.W., Aschner M. (2015). Mitochondrial Redox Dysfunction and Environmental Exposures. Antioxid. Redox Signal..

[35] Devarie-Baez N.O., Silva Lopez E.I., Furdui C.M. (2016). Biological chemistry and functionality of protein sulfenic acids and related thiol modifications. Free Radic. Res..

[36] Sena L.A., Chandel N.S. (2012). Physiological Roles of Mitochondrial Reactive Oxygen Species. Mol. Cell.

[37] Holmila R.J., Vance S.A., Chen X., Wu H., Shukla K., Bharadwaj M.S., Mims J., Wary Z., Marrs G., Singh R. (2018). Mitochondria-targeted Probes for Imaging Protein Sulfenylation. Sci. Rep..

[38] Kleinauskas A., Rocha S., Sahu S., Sun Y.P., Juzenas P. (2013). Carbon-core silver-shell nanodots as sensitizers for phototherapy and radiotherapy. Nanotechnology.

[39] Swanner J., Mims J., Carroll D.L., Akman S.A., Furdui C.M., Torti S.V., Singh R.N. (2015). Differential cytotoxic and radiosensitizing effects of silver nanoparticles on triple-negative breast cancer and non-triple-negative breast cells. Int. J. Nanomed..

[40] Nguyen K.C., Seligy V.L., Massarsky A., Moon T.W., Rippstein P., Tan J., Tayabali A.F. (2013). Comparison of toxicity of uncoated and coated silver nanoparticles. J. Phys. Conf. Ser..

[41] Swanner J., Fahrenholtz C.D., Tenvooren I., Bernish B.W., Sears J.J., Hooker A., Furdui C.M., Alli E., Li W., Donati G.L. (2019). Silver nanoparticles selectively treat triple-negative breast cancer cells without affecting non-malignant breast epithelial cells in vitro and in vivo. FASEB BioAdv..

[42] Yamamori T., Yasui H., Yamazumi M., Wada Y., Nakamura Y., Nakamura H., Inanami O. (2012). Ionizing radiation induces mitochondrial reactive oxygen species production accompanied by upregulation of mitochondrial electron transport chain function and mitochondrial content under control of the cell cycle checkpoint. Free Radic. Biol. Med..

[43] Gurunathan S., Qasim M., Park C., Yoo H., Kim J.H., Hong K. (2018). Cytotoxic Potential and Molecular Pathway Analysis of Silver Nanoparticles in Human Colon Cancer Cells HCT116. Int. J. Mol. Sci..

[44] Cameron S.J., Hosseinian F., Willmore W.G. (2018). A Current Overview of the Biological and Cellular Effects of Nanosilver. Int. J. Mol. Sci..

[45] Jezek J., Cooper K.F., Strich R. (2018). Reactive Oxygen Species and Mitochondrial Dynamics: The Yin and Yang of Mitochondrial Dysfunction and Cancer Progression. Antioxidants.

[46] Wu H., Lin J., Liu P., Huang Z., Zhao P., Jin H., Ma J., Wen L., Gu N. (2016). Reactive oxygen species acts as executor in radiation enhancement and autophagy inducing by AgNPs. Biomaterials.

[47] Wu H., Lin J., Liu P., Huang Z., Zhao P., Jin H., Wang C., Wen L., Gu N. (2015). Is the autophagy a friend or foe in the silver nanoparticles associated radiotherapy for glioma?. Biomaterials.

[48] Zheng Q., Yang H., Wei J., Tong J.L., Shu Y.Q. (2013). The role and mechanisms of nanoparticles to enhance radiosensitivity in hepatocellular cell. Biomed. Pharmacother..

[49] Che B., Luo Q., Zhai B., Fan G., Liu Z., Cheng K., Xin L. (2017). Cytotoxicity and genotoxicity of nanosilver in stable GADD45alpha promoter-driven luciferase reporter HepG2 and A549 cells. Environ. Toxicol..

[50] Rosario F., Hoet P., Santos C., Oliveira H. (2016). Death and cell cycle progression are differently conditioned by the AgNP size in osteoblast-like cells. Toxicology.

[51] Eom H.J., Choi J. (2010). p38 MAPK activation, DNA damage, cell cycle arrest and apoptosis as mechanisms of toxicity of silver nanoparticles in Jurkat T cells. Environ. Sci. Technol..

[52] Loutfy S.A., Al-Ansary N.A., Abdel-Ghani N.T., Hamed A.R., Mohamed M.B., Craik J.D., Eldin T.A., Abdellah A.M., Hussein Y., Hasanin M.T. (2015). Anti-proliferative Activities of Metallic Nanoparticles in an in Vitro Breast Cancer Model. Asian Pac. J. Cancer Prev..

[53] Kawamura K., Qi F., Kobayashi J. (2018). Potential relationship between the biological effects of low-dose irradiation and mitochondrial ROS production. J. Radiat. Res..

[54] Kam W.W., Banati R.B. (2013). Effects of ionizing radiation on mitochondria. Free Radic. Biol. Med..

[55] Gorrini C., Harris I.S., Mak T.W. (2013). Modulation of oxidative stress as an anticancer strategy. Nat. Rev. Drug Discov..

[56] Menon S.G., Goswami P.C. (2007). A redox cycle within the cell cycle: Ring in the old with the new. Oncogene.

[57] Sarsour E.H., Kumar M.G., Chaudhuri L., Kalen A.L., Goswami P.C. (2009). Redox control of the cell cycle in health and disease. Antioxid. Redox Signal..

[58] Ma W., Jing L., Valladares A., Mehta S.L., Wang Z., Li P.A., Bang J.J. (2015). Silver nanoparticle exposure induced mitochondrial stress, caspase-3 activation and cell death: Amelioration by sodium selenite. Int. J. Biol. Sci..

[59] Maurer L.L., Meyer J.N. (2016). A systematic review of evidence for silver nanoparticle-induced mitochondrial toxicity. Environ. Sci. Nano.

[60] Takenaka S., Karg E., Roth C., Schulz H., Ziesenis A., Heinzmann U., Schramel P., Heyder J. (2001). Pulmonary and systemic distribution of inhaled ultrafine silver particles in rats. Environ. Health Perspect..

[61] Marchi E., Vargas F.S., Teixeira L.R., Acencio M.M., Antonangelo L., Light R.W. (2005). Intrapleural low-dose silver nitrate elicits more pleural inflammation and less systemic inflammation than low-dose talc. Chest.

[62] Arsenopoulou Z.V., Taitzoglou I.A., Molyvdas P.A., Gourgoulianis K.I., Hatzoglou C., Zarogiannis S.G. (2017). Silver nanoparticles alter the permeability of sheep pleura and of sheep and human pleural mesothelial cell monolayers. Environ. Toxicol. Pharmacol..

